# Emission characteristics of cellulosic jet biofuel blend under laminar and turbulent combustion

**DOI:** 10.1186/s13068-023-02439-4

**Published:** 2023-12-19

**Authors:** Ziyu Liu, Zhichao Wang, Xiaoyi Yang

**Affiliations:** 1 School of Energy and Power Engineering, Beihang University, 37 Xueyuan Rd, Beijing, 100191 People’s Republic of China; 2 School of Aeronautic Science and Engineering, Beihang University, 37 Xueyuan Rd, Beijing, 100191 People’s Republic of China

**Keywords:** Bunsen burner, Engine, Emission characteristics, PM, NOx

## Abstract

Alternative biofuels have the potential to reduce greenhouse gas emissions and particulate matter due to free of aromatics compared to traditional petroleum-based aviation fuel. The potential mitigating emission of hydrothermal-condensation-hydrotreating jet biofuel (HCHJ) derived from agriculture residue was investigated. The effects of aviation biofuel components, blend ratio and equivalent ratio on emission characteristics were conducted by Premixed Pre-evaporated Bunsen burner (PPBB) for laminar combustion and ZF850 jet engine for turbulent combustion. In compositions, HCHJ had a higher concentration of cycloparaffins (mostly in C8–C10) while petroleum-based aviation fuel (RP-3) had a higher concentration of alkylbenzenes (mostly in C8–C11). In laminar combustion, HCHJ and both 50% blend HCHJ appear no unburned hydrocarbon (UHC) due to low aromatics content and no sulfur in the biofuel. Moreover, there were no significant differences in NO and NO_2_ concentration for HCHJ and HCHJ blends. In turbulent combustion, HCHJ blends and RP-3 were compared engine emissions at various state points. Considering all complex effects of fuel and combustion environment, HCHJ blend had a noticeable reduction in PM_2.5_ emissions in comparison with RP-3 due to their lower aromatics and sulfur content. As HCHJ is similar to RP-3 in C/H ratio, density and heat value and the different aromatics contents have different tendencies to generate PM_2.5_ at different condition, PM_2.5_ emission is not only related with the total aromatic content and individual aromatic structure but also the combustion environment at thrust setting and coexisting pollutants including NOx and UHC emissions. CO and NOx emission indicated that both of turbulent state and fuel type influence emissions. HCHJ blend can be benefit for PM_2.5_ reduction and combustion efficiency growth. PM_2.5_ reduction can be obtained 77.5% at 10% HCHJ blend and 9.5% at 5% HCHJ blend while combustion efficiency can be obtained 0.05% at 5% HCHJ blend and 0.36% at 10% HCHJ blend through all thrust output.

## Introduction

Alternative biofuels have been confirmed to reduce greenhouse gas emissions and benefit for clean sky [1–3]. However, it is still important to determine whether engine emissions are affected by substituting alternative fuels for traditional petroleum-based sources. Moreover, as particulate matter (PM) emissions from aircraft turbine engines can deteriorate air quality and contribute to climate change [4, 5], PM is a growing concern due to its potential impact on both climate change and local air quality [6]. Alternative aviation fuels have a potential to reduce emissions due to their lower aromatics and sulfur content [7], and the use of sustainable aviation fuels (SAFs) is expected to reduce GHGs emission and PM emissions [8] until advanced engines with lower emissions become available. Therefore, it is crucial to understand emission characteristics of engines when burning alternative fuels.

The blend biofuels with conventional jet fuel have noticed an improvement in engine performance and the reduction in emission level [9, 10]. The effects of fuel blends on emission have been found different at low load power and high load power. The reduction in emission is a function of the jet engine power setting with the largest reductions usually observed at low power conditions. Besides GHGs reduction, SAFs blend may have a positive impact on the reduction of particulate emissions [11, 12]. Alternative jet fuel derived from hydro-treated ester and fatty acid (HEFA) present that emission indices of NOx, CO and UHC were as same as conventional jet fuel but PM emission was quite lower [13]. SAFs blend led to a reduction of 70% in PM mass compared to traditional petroleum-based jet fuel by a CFM56-5C4 engine [14–16]. The highest soot reductions were observed at lower power settings. The soot emission showed a good correlation to the hydrogen content of the fuel. The application of ternary blends with beneficial emission properties has been demonstrated on PW4158 engines as well [17]. Size distribution of PM has been discovered the relationship with fuel flow and fuel composition by a CFM56-2C1 engine [13]. The PM mass emissions varied depending on fuel flow, fuel type, and sampling temperature with a characteristic U-shaped curve of PM mass emissions with respect to fuel flow [18]. At low fuel flow corresponding to low engine power, particle number and volume size distributions contained a single mode, whereas a bimodal distribution was observed at higher engine power. The black carbon emissions were found to exponentially increase with engine power. The use of two neat alternative fuels reduced PM number emission index by a median value of 70–73% as compared to JP-8 across all power conditions tested. In piston engine, strategies of fuel injection can also contribute on PM formation [19, 20]. The higher NOx emissions were acquired across all biodiesel blend fuels due to the occurrence of bonded oxygen with the absence of aromatics in the biofuels [21, 22]. CeO_2_ nanoparticle blends were used for further reduction of PM [23]. HEFA alternative fuel derived from used cooking oil (UCO) was performed PM reduction in 16 different blends by a GTCP85 aircraft auxiliary power unit (APU) [24]. The reductions in PM were found to be greater with increasing fuel hydrogen content and the average reduction in PM number-based emissions was about 35% while that for mass-based emissions was about 60%.

By a CFM56-7B engine burning different blends of Jet A-1 and HEFA [7], HEFA blend with 32% was observed a reduced emission of elemental carbon mass in the range of 50% to 60% at low power settings. The PM emission indices were reduced most markedly by 70% in terms of PM mass and 60% in terms of PM number at idle engine power [16]. The relative reduction of PM emissions decreased with the increasing thrust. SAF blends reduced the PM emissions from the standardized landing and take-off cycle by 20% in terms of PM mass and 25% in terms of PM number. Elser [8] conducted a study on a CFM56-7B turbofanto investigate the link between the chemical composition and optical properties of PM at the engine exit plane. Using different fuel blends with conventional Jet A-1 across a wide range of power settings, they found that the absorption and scattering coefficients, as well as the PM mass, increased with thrust at both measurement wavelengths (532 and 870 nm). However, the use of HEFA blends induced a significant decrease in PM mass and optical coefficients at all thrust levels. By assessing the multiblend Jet A-1 [17], the emission of particle mass is reduced by about 29% and the number of emitted particles is reduced by around 37% based on landing-and-take-off cycle. Moore [25] statistically analyzes the impact of jet fuel properties on aerosols emitted through CFM56-2-C1 engines by burning 15 different aviation fuels, and the results indicated that aromatic and sulfur content in the fuel affect significantly the volatile aerosol fraction, which dominates the variability of the number and volume emissions indices (EIs) over all engine powers. Meanwhile, the naphthalene content of the fuel determines the magnitude of the EI of particle mass. Linear regression coefficients were evaluated according to each aerosol EI in terms of these properties, engine fuel flow rate, and ambient temperature. The results were found that reduction of both fuel sulfur content and naphthalene to near-zero levels would lead to roughly a tenfold decrease in aerosol number emitted per kilogram of fuel burned. SAFs blend including FT-SPK, HEFA-SPK, HFS-SIP and ATJ were confirmed to reduce PM reduction due to free of cycloparaffin, aromatic and sulfur. Therefore, the efforts to reduce aircraft emissions should shift towards low-aromatic, low-sulfur alternative jet fuels [26, 27]. Concerning NOx emissions, no significant differences were found in blend fuels [5, 28]. FT fuel free of aromatics displayed slightly higher NOx values [28] and slight differences were observed in NO and NO_X_ emissions due to the differences in H/C ratio of the fuels. NO and NO_2_ is a characteristic of the engine and combustor design and a function of engine operating conditions [5].

Recently, there has been a growing interest in using cellulosic agriculture residue for bio-jet fuel due to broad feedstocks with low sulfur content [1, 29, 30]. Cellulosic hydrothermal-condensation-hydrotreating jet fuel (HCHJ) has emerged as a potential solution for producing hydrocarbon jet biofuel through hydrotreatment of oxygenated intermediates derived from aldol condensation of furfural and levulinic acid, which can be produced by hydrothermal decomposition of lignocellulosic biomass [1]. The difference between HCHJ and the other SAFs lies in abundant cycloparaffins in HCHJ. The development of sustainable aviation fuels is crucial to reduce greenhouse gas emissions and particulate matter. From the above discussion, it is evident that most of the alternative biofuel demonstrates a significant potential as an additive fuel for the optimization of the gaseous emissions on turbine engine. PM formation is a complex relationship with fuel composition, and emission is a function of the jet engine power setting, and thus it is essential to explore HCHJ for mitigating PM. This study aims to examine how the composition and properties of blend fuels impact emissions under laminar combustion by a premixed pre-evaporated Bunsen burner and turbulent combustion by ZF850 turbine engine. Emissions data including PM, CO, UHC, and NOx, were collected at different thrust settings to gain insight into expected in-flight emissions.

## Materials and methods

### Compositions and properties

The compositions of HCHJ and RP-3 jet fuel were investigated using a GC–MS system (Agilent 7890A-5975C) with an HP-5 capillary column at a split ratio of 20:1. The oven was controlled starting at 50 °C for 2 min and ramped to 175 °C at 5 °C/min for 2 min, and then ramped to 300 °C at 2 °C/min for 2 min. The mass spectrometer scan ranged from m/z 30 to m/z 800. The injector and detector temperatures were 280 °C and 150 °C. The carbon distribution and classification distribution were identified by compositions using NIST. Fuel properties, including heat value and density, were investigated based on ASTM methods.

### Premixed pre-evaporated Bunsen burner experiment

**Fig. 1:**
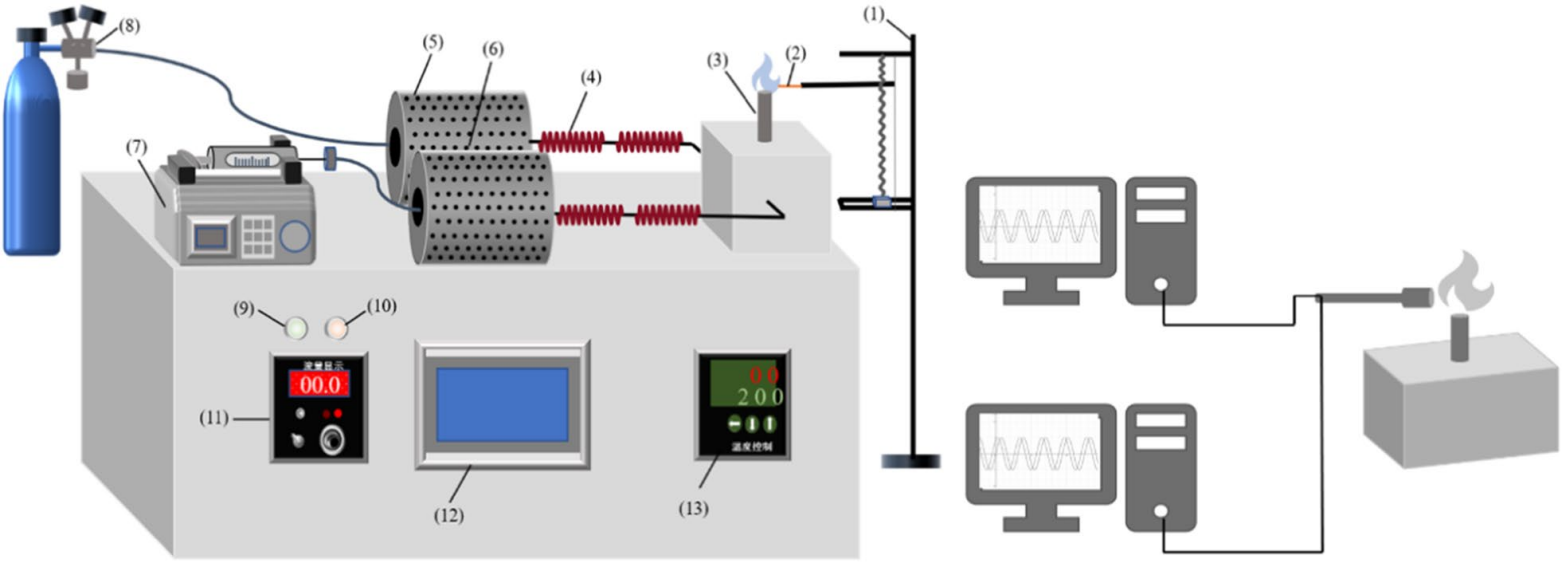
Premixed pre-evaporated Bunsen burner setup

The premixed pre-evaporated Bunsen burner (PPBB) consists of three units, as shown in Fig. 1, including the pre-evaporated heating tube, premixed chamber, and Bunsen burner. Fuel is pre-evaporated by passing through the heating fuel tube, which can be controlled at a temperature in the range of 20 ℃–450 ℃. Similarly, air is heated by passing through the heating air tube. The pre-evaporated jet fuel and heated air are then injected into the premixed chamber, where they are mixed thoroughly. The resulting mixture is then fed into the Bunsen burner, where a stable laminar flame is created by controlling the flow rate and the ratio of fuel to air. For investigating the different positions of the flame, test probes were controlled by a three-dimensional displacement instrument. The emission signal was recorded per a second. Carbon dioxide (CO_2_, ± 0.01%) and unburned hydrocarbons (CH_4_,  ± 1 ppm) were investigated by a nondispersive infrared sensor, while carbon monoxide (CO,  ± 1 ppm) and nitrogen oxides (NO_*x*_,  ± 0.1 ppm) were measured by electrochemical sensors. Furthermore, the study examined PM_2.5_ particulate matter using a laser particle analysis instrument (PM_2.5_,  ± 0.001mg/m^3^).

### Engine experiment

**Fig. 2:**
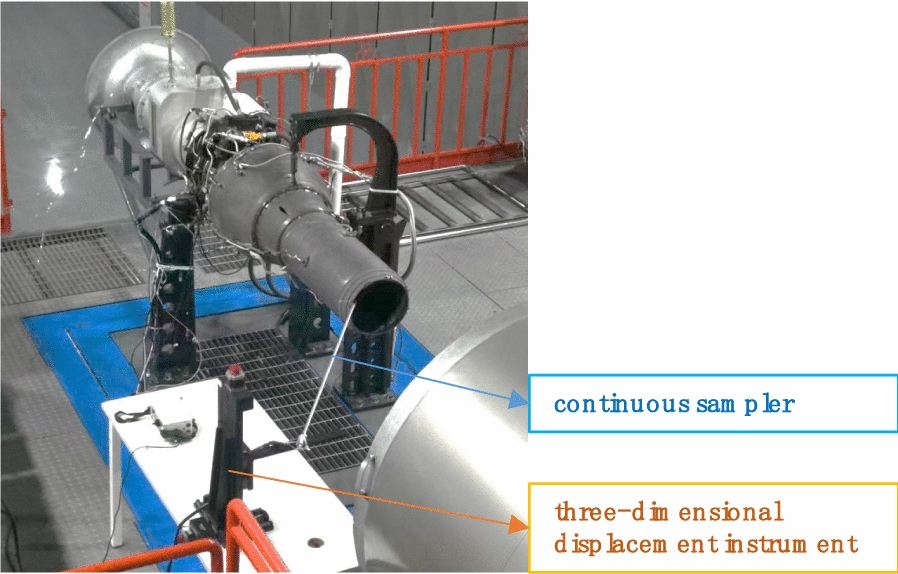
ZF850 Engine test rig

ZF850 jet engine is characterized with low fuel consumption and high flight contour, which is appropriate for assessing the fuel effects on engine emission. The fuel is supplied to the engine by a fuel pump and its flow is controlled by a desired engine speed signal. The data acquisition system communicates with a high-speed microprocessor circuit that remotely communicates with the engine control unit to control the desired engine speed (Fig. 2).

ZF850 jet engine was operated at the different state points including 48% (10,500 r/min), 55% (12,100 r/min), 72% (15,840 r/min), 82% (18,040), 90% (19,800 r/min), 95% (20,900 r/min), 97% (21,340 r/min). The engine emissions were recorded by on-line the data acquisition system as PPBB per second. The fuel-mass specific emissions index (EI) was used to present emissions concentrations normalized to fuel flow, which were recorded per 0.08 s. Emissions in exhaust stream can represent the degree of incomplete combustion and hence a loss of potential heat release including UHC, CO and PM_2.5_, which were all involved for calculation of combustion efficiencies as follow:

$$\mathrm{Combustion\, efficiency}= \frac{1-({\mathrm{EI}}_{\mathrm{UCH}}\times {\mathrm{HV}}_{\mathrm{UCH}}+{\mathrm{EI}}_{\mathrm{CO}}\times {\mathrm{HV}}_{\mathrm{CO}}+{\mathrm{EI}}_{\mathrm{PM}}\times {\mathrm{HV}}_{\mathrm{PM}}) }{{\mathrm{HV}}_{\mathrm{fuel}}}$$

## Results and discussion

### Compositions characteristics

**Fig. 3:**
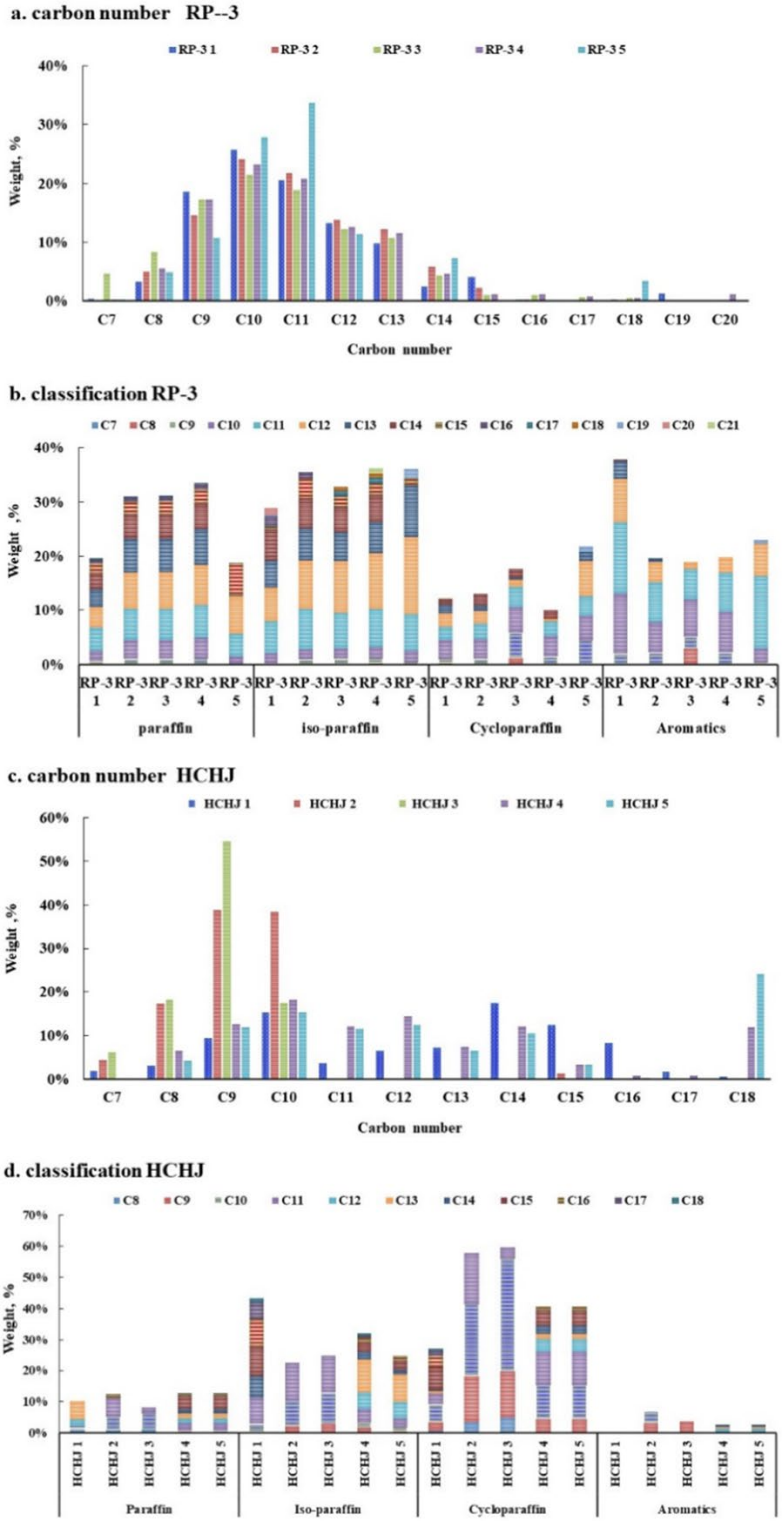
Carbon distribution and classification distribution

Traditional jet fuels are composed of different hydrocarbon groups, including *n*-paraffin, iso-paraffin, cycloparaffin, and aromatics with similar carbon number distributions. Carbon distribution of fuels covers from C7 to C20. RP-3 jet fuels display a normal distribution from C8 to C14 centered on C10 and C11, while HCHJ jet fuels display a different distribution compared with RP-3, which are abundant in C8, C9, and C10 but less C11, C12, C16, which could be attributed the refining process. Moreover, HCHJ has a higher percentage of hydrocarbons with a carbon number of C18 compared to RP-3, given in Fig. 3.

From the view of classification distribution, RP-3 jet fuels contain high alkylbenzenes (mostly in C8–C11), while HCHJ jet fuels contain high cycloparaffin (mostly in C8–C10). In RP-3, alkylbenzenes can make up 20.56% of the composition (mostly in C10), while HCHJ has almost no alkylbenzenes but has a higher percentage of cycloparaffin at 57.93% compared to RP-3 18.89%. Although HCHJ fuels and RP-3 fuels present significant distinction in carbon distribution and classification, HCHJ fuels and RP-3 fuels present similar C/H ratios (5.8–5.9), densities (800–810 kg/m^3^), and heat values (43.5–43.9 MJ/kg).

### Emission characteristics in PPBB

**Fig. 4:**
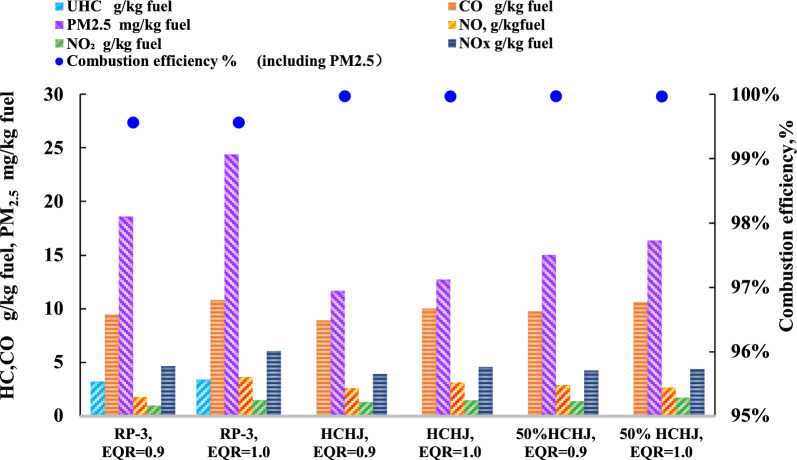
Comparison of the emission of RP-3 and HCHJ

The effects of fuel components with different equivalent ratios (EQR) on combustion emissions were studied on the PPBB test rig, as shown in Fig. 4.

In the respect of EQR effects, all EIs of CO, UHC and PM increase when EQR change from 0.9 to 1.0 regardless of RP-3, HCHJ or HCHJ 50%, which comply with combustion rules that moderate excess oxygen could benefit the reduction of emission and increase of combustion efficiency. In comparison with fuel components effects, EIs of CO, UHC and PM follow the order RP-3 > HCHJ 50% > HCHJ at same EQR. Unburned hydrocarbon (UHC) was not detected in the combustion process both of 50% blend cellulosic jet oil and cellulosic jet oil, which could be attributed to lower aromatics content and no sulfur in biofuel. NOx emissions are complex which are related with the combustion temperature. NO increase with EQR from 0.9 to 1.0 which may be deduced the higher combustion temperature at 1.0 EQR regardless RP-3, HCHJ or HCHJ 50%. There are no obvious different both of NO and NO_2_ concentration between HCHJ and HCHJ 50%. RP-3 presents lower NOx El than HCHJ or HCHJ 50% at 0.9 EQR while present higher NOx El than HCHJ or HCHJ 50% at 1.0 EQR. For at 0.9 EQR, the reason could be that more UHC and PM in RP-3 combustion emission leads to NOx reduction. HCHJ fuel and HCHJ blend fuel appear higher combustion efficiency than RP-3 at 0.9 EQR to 1.0 EQR.

**Fig. 5:**
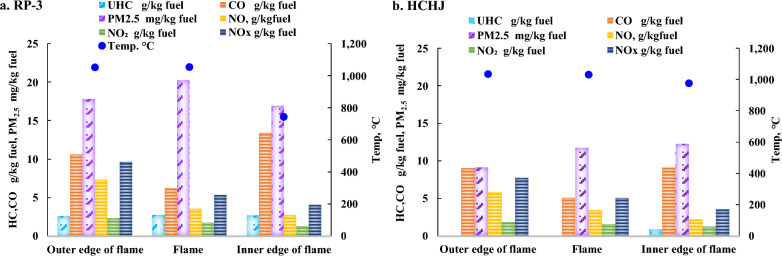
Comparison of the emission index at various flame position

For discovering the combustion process, the emission index of RP-3 and HCHJ was compared at various flame position, given in Fig. 5. It is found that the PM_2.5_ showed a gradually decrease from inner edge of flame to outer edge of the flame. At the inner edge of flame, the temperature is the lowest but with the largest concentration of PM_2.5_. The soot particles generated at the inner cone corner continue to participate in combustion as they move towards the flame surface, and the concentration of PM_2.5_ decreases combined with the concentration of CO increases. This result is coincidence with the formation mechanism of carbon smoke in the combustion process. With temperature increase near to combustion, fuel composition was firstly pyrolyzed and broken into small molecules of hydrocarbons, which then form a single ring of aromatic hydrocarbons and further grow into polycyclic aromatic compounds. Polycyclic aromatic compounds can be considered as the initial particles of soot nucleation. After the formation of the initial particles, soot particles may merge into larger soot particles by colliding with other initial particles of soot in the oxygen deficiency condition. With moving into flame, particle matter may react with oxygen to CO or CO_2_.

From Fig. 5, UHC can only be detected at inner edge of flame for HCHJ while UHC can be detected even at outer edge of flame. Both of RP-3 and HCHJ present the highest CO concentration at the inner edge and the lowest of CO concentration was found at the surface of flame. NOx all showed an obvious increase from inner edge to outer edge of the flame. As the increase of flame temperature leads to more NOx formation which moved outward with combustion gas, the highest NOx has been investigated at the outer edge of flame. NO increase obviously while NO_2_ increase scarcely. NOx emissions are induced by three mechanisms namely thermal, fuel and prompt. Fuel NOx formation can be negligible due to less nitrogen in RP-3 and free of nitrogen in HCHJ. For prompt NOx formation, it can also be negligible due to lower fuel air ratio in engine control. Therefore, NOx formation is mainly derived from thermal mechanism of N_2_ oxidation. NOx can be reduced to N_2_ by PM_2.5_, UHC and CO.

### Emission characteristics in engine

**Fig. 6:**
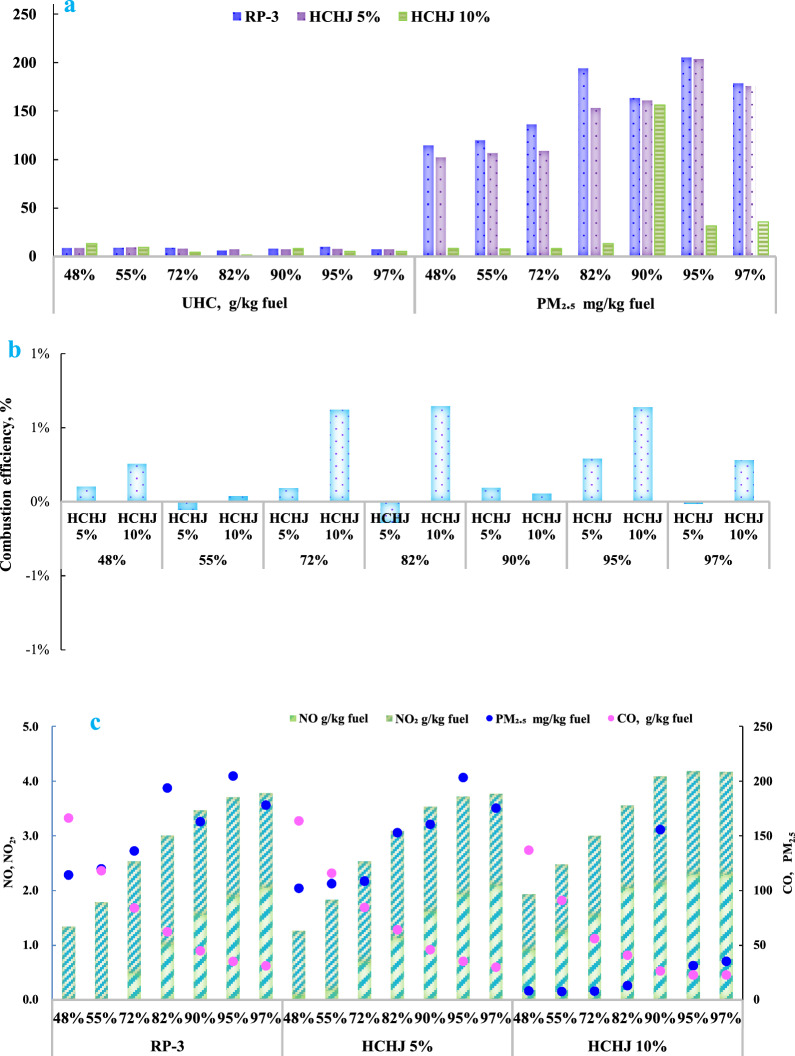
Comparison of the emission index. **a** UHC and PM_2.5_; **b** combustion efficiency; **c** NO, NO_2_,NOx,CO,PM_2.5_

The impact of HCHJ blend on engine performance was studied at various engine speeds from 48% thrust to 97% thrust, as depicted in Fig. 6. The fuel–air ratios were adjusted in compliance with engine speed in combination with inlet temperature and inlet pressure. At low power loads, reducing the fuel/air ratio could reduce the EI_UHC_, but this would also lower the combustion temperature which could extend the timescale of complete combustion. At high power loads, increasing the fuel/air ratio could increase the EI_UHC_ emissions due to higher fuel consumption, but the higher combustion temperature could accelerate combustion reactions. Considering all these complex effects, EI_UHC_ showed emission peaks at 48% thrust and 95% thrust, with lower emissions in the moderate range of 55–90% thrust.

EIPM_2.5_ is characterized with a complex relationship of thrust, and two types of jet fuel present different emission characteristics. The emission characteristics of EIPM_2.5_ can help understand how individual components in the jet fuel could influence PM_2.5_ formation. PM_2.5_ derived from gas turbine emission has been investigated the relationship with fuel properties including hydrogen content [4, 31], hydrogen-to-carbon ratio [27, 32], total aromatic content, threshold sooting index [33, 34]. HCHJ blend could be benefit for PM_2.5_ reduction. Through all thrust output, PM_2.5_ reduction can be obtained 77.5% at 10% HCHJ blend and 9.5% at 5% HCHJ blend throughout the entire range of thrust output setting. Moreover, combustion efficiency can be improved 0.05% at 5% HCHJ blend and 0.36% at 10% HCHJ blend. However, the effects of fuel blends on emission have been found different at various thrust output settings. PM_2.5_ reduction changed with thrust output settings and the largest reductions were observed at low power conditions, which is consistent with the previous research [9]. At various thrust output settings due to change in FAR and fuel flow, the turbulent state in combustor presents significantly distinction. In general, combustor is designed the best turbulent state at cruise. Hence, at low power conditions, the inlet temperature and inlet pressure with low FAR usually lower then at cruise conditions. Although lower FAR ratios could improve combustion emission but extend the timescale of combustion completeness due to the lower combustion temperature at low thrust output, which could enhance PM_2.5_ formation at low thrust output settings. At various thrust output settings, PM_2.5_ emission further confirmed that both of turbulent state and fuel effect influence PM_2.5_ formation. There is clear indication that PM_2.5_ formation in all conditions is attributed with combustion mechanism and turbulent state. As the different aromatics contents have different tendencies to generate PM_2.5_ at different condition, PM_2.5_ is not only related with the total aromatic content and individual aromatic structure but also the combustion environment at thrust setting and coexisting pollutants including NOx and UHC emissions.

For EI_CO_ and EI_NOx_, there was almost no obvious difference between RP-3 and HCHJ blend jet biofuel. NOx emission is the only emission gas which is noticeable at high load due to high temperature. EI_NOx_ indicates a very strong influence of power settings, which can be attributed the effects of flame temperature, whereas NOx concentrations at high power load can be much higher than for those at low power load. NOx emissions increase gradually with engine speed due to the higher temperatures generated at high speeds, leading to increased NOx pollution. On the other hand, CO emissions decrease with speed increasing. This suggests that nitrogen oxide production increases with temperature, while carbon monoxide production decreases. In general, an increase in combustor-inlet temperature and pressure leads to a decrease in carbon monoxide and unburned hydrocarbon emissions, while the opposite is true for nitrogen oxides (NOx).

NOx formation can be ascribed to three mechanisms including thermal formation, fuel formation and prompt formation. Fuel formation can be negligible for no nitrogen in HCHJ fuel and less nitrogen in RP-3 fuel. Prompt formation is related by the radical concentration of hydrocarbon which can oxide N_2_ to form NOx and meanwhile NOx can be reduced to N_2_ by UHC and CO. NOx formation through prompt pathway is complex which is controlled by turbulent flow, temperature peak distribution, and fuel type. Thermal formation is mainly controlled by temperature peak distribution in combustor, where flame temperatures increase with thrust output growth and lead to more NOx formation. The emission results of EI_CO_ and EI_NOx_ indicated that both of turbulent state and fuel type influence emission of CO and NOx as same as PM_2.5_.

## Conclusion

For laminar flame in PPBB test, HCHJ biofuel blend presents obviously PM_2.5_ reduction and UHC reduction. For turbulent flame on engine test, PM_2.5_ is not only related with fuel composition but also combustion environment at thrust setting. The emission of EIs of CO and NOx had a strong relationship with thrust setting, and CO and NOx present negative correlation with high NOx concentrations corresponding to lower CO concentrations.

## Data Availability

The datasets generated or analyzed during this study are available from the corresponding author on reasonable request.

## Abbreviations

UHC: Unburned hydrocarbon
PM: Particulate matter
SPK: Synthesized paraffinic kerosine
FT: Fischer–Tropsch
HEFA: Hydroprocessed esters and fatty acids
HFS-SIP: Hydroprocessed fermented sugars synthesized iso-paraffins
HCHJ: Hydrothermal-condensation-hydrotreating jet fuel (HCHJ)
ATJ: Alcohol-To-Jet
EQR: Equivalent ratios
RP-3: Traditional jet fuel
SAFs: Sustainable aviation fuels
PPBB: Premixed pre-evaporated Bunsen burner
